# Eruptive pruritic papular porokeratosis responsive to JAK1 inhibition

**DOI:** 10.1016/j.jdcr.2026.05.023

**Published:** 2026-05-15

**Authors:** Caroline Echeandia-Francis, William E. Damksy, Marc E. Grossman, Keith A. Choate

**Affiliations:** a Department of Dermatology, Yale University School of Medicine, New Haven, Connecticut; b Department of Pathology, Yale University School of Medicine, New Haven, Connecticut; c Department of Genetics, Yale University School of Medicine, New Haven, Connecticut

**Keywords:** eruptive pruritic papular porokeratosis, inflammatory dermatosis, JAK inhibition, porokeratosis, RNA in situ hybridization, upadacitinib

Porokeratosis is an epidermal differentiation disorder characterized by papules or plaques encircled by a collar of peripheral scale (cornoid lamella). Localized variants include porokeratosis of Mibelli, linear porokeratosis, porokeratosis palmaris et plantaris disseminata, and punctate porokeratosis, whereas generalized forms include disseminated superficial actinic porokeratosis (DSAP) and disseminated superficial porokeratosis. Eruptive pruritic papular porokeratosis is a rare variant of porokeratosis characterized by the appearance of pruritic inflammatory papules, often at the side of prior porokeratosis lesions. Topical therapies include 5-fluorouracil, topical corticosteroids, keratolytics, topical retinoids, imiquimod 5%, and compounded topical cholesterol–lovastatin.^1^ Here, we present a case of eruptive pruritic papular porokeratosis, characterize the inflammatory infiltrate associated, and demonstrate efficacy of Janus kinase (JAK) inhibition in reducing lesion thickness and pruritus this relatively rare variant.

An 80-year-old man presented in September 2024 with erythematous annular and round plaques, most with peripheral and central clearing on his scalp, chest, back, and extremities (Fig 1). There were isolated plaques with confluent scale, and he reported that these lesions became evident in early March 2021, shortly after receiving 2 doses of the Moderna COVID vaccination (first dose: January 28, 2021; second dose: February 25, 2021) and attributed their onset to a cutaneous reaction caused by the vaccine. The lesions were intensely pruritic, and excoriation resulted in widespread hemorrhagic crusting. His sleep and quality of life were severely affected. He had a personal, but not a family, history of eczematous dermatitis. For several months, compounded topical cholesterol-lovastatin ointment was applied from the knees to the ankles and weekly subcutaneous dupilumab injections were administered without benefit.

**Fig 1:**
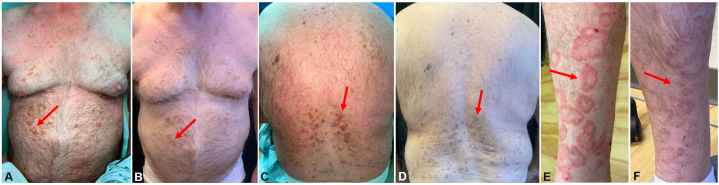
Torso, back, and right distal lower extremity before and after 4 wk of upadacitinib treatment. **A, C,** and **E,** Torso, back, and right distal lower extremity prior to treatment with upadacitinib, showing multiple papules and plaques of porokeratosis with pronounced erythema. The lower extremity had previously been treated with several months of topical cholesterol–lovastatin ointment. **B, D,** and **F,** Torso, back, and right distal lower extremity after 4 wk of upadacitinib treatment, demonstrating a significant reduction in both erythema and lesion thickness.

Two biopsies obtained from the right back and right arm demonstrated cornoid lamellae, consistent with porokeratosis (Fig 2). Both specimens also showed a superficial perivascular and focally lichenoid lymphohistiocytic inflammatory infiltrate (Fig 2), a finding most often found in eruptive papular pruritic porokeratosis. RNA in situ hybridization (RISH) revealed expression of interferon-gamma and interleukin 13, while psoriasis-associated biomarkers interleukin 17 A and nitric oxide synthase 2 were absent. The diagnosis supported a diagnosis of eruptive pruritic papular porokeratosis.

**Fig 2:**
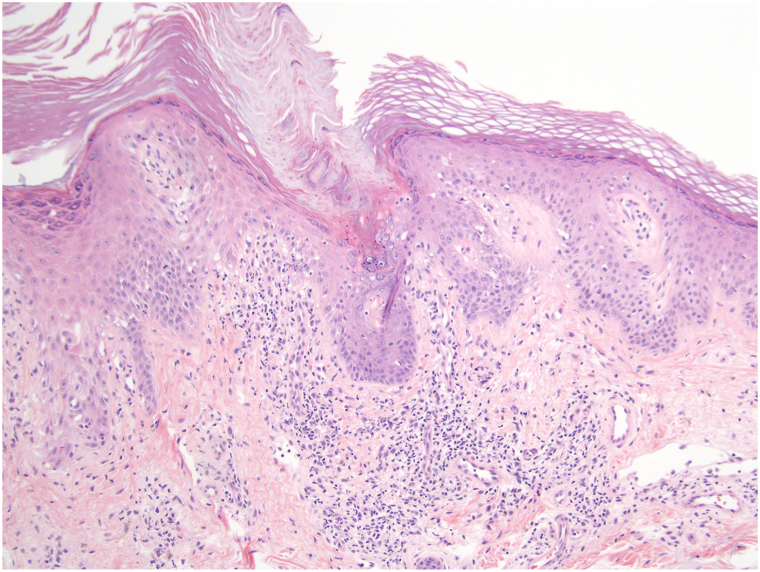
Histologic features of porokeratosis (H&E). A characteristic cornoid lamella is present, composed of a thin column of parakeratosis overlying focal loss of the granular layer and dyskeratotic keratinocytes. The adjacent epidermis shows mild acanthosis. A superficial perivascular and interface lymphocytic inflammatory infiltrate is noted in the underlying dermis, most prominent beneath the cornoid lamella.

Given the lack of response to dupilumab and the presence of interferon-gamma expression, the patient was transitioned to upadacitinib, a JAK1 inhibitor, at a dose of 15 mg daily, and dupilumab was discontinued.

After 1 month of treatment, the patient reported marked improvement in pruritus, along with a reduction in erythema and thickness of the inflammatory component of the lesions (Fig 1). He continued upadacitinib 15 mg daily and resumed twice-daily application of topical cholesterol-lovastatin to the lower extremities.

The clinical presentation in this case is most consistent with eruptive pruritic papular porokeratosis. Hallmark features supporting this diagnosis include the sudden development of widespread, papular lesions, severe pruritus, and histopathologic evidence of inflammation within porokeratotic lesions. In contrast to classic DSAP, which follows a chronic, indolent course and is often minimally symptomatic,^2^ eruptive pruritic papular porokeratosis is defined by its inflammatory phenotype and symptom burden.^2^, ^3^, ^4^ Although inflammatory exacerbations have been reported in patients with longstanding DSAP following systemic triggers such as viral infection, medication exposure, or chemotherapy,^5^ these cases are increasingly recognized as falling within the spectrum of eruptive pruritic papular porokeratosis rather than representing simple progression of DSAP.

The inflammatory and eczematous features observed in this patient are best interpreted as integral to the pathophysiology of eruptive pruritic papular porokeratosis rather than as a superimposed or secondary dermatitis given confinement to lesional skin. Prior reports of inflammatory porokeratosis variants describe spongiotic and lymphocytic infiltrates within porokeratotic lesions,^4^^,^^6^, ^7^, ^8^ supporting the concept that epidermal inflammation may be a defining feature of this subtype.

The immune mechanisms underlying eruptive pruritic papular porokeratosis remain poorly defined.^9^ While DSAP has been associated with pathogenic variants in genes within the mevalonate kinase pathway,^10^ it is not classically associated with a consistent inflammatory cytokine signature. The inflammatory infiltrates described in eruptive pruritic papular porokeratosis appear heterogeneous and are thought to reflect immune dysregulation rather than primary autoimmune disease.^6^ In this case, the temporal association between symptom onset and COVID-19 vaccination may represent an immune-activating trigger in a predisposed host; however, this association should not be interpreted as evidence of causality. Viral infections and vaccinations have been reported to precipitate flares of inflammatory dermatoses, and similar mechanisms may contribute to disease expression in eruptive pruritic papular porokeratosis.

The patient’s therapeutic response further supports an inflammatory-driven phenotype. Despite evidence of interleukin 13 expression, treatment with dupilumab was ineffective, suggesting that T helper type 2 signaling alone may not be the dominant driver of inflammation in this condition. In contrast, treatment with the JAK1 inhibitor upadacitinib resulted in marked improvement in pruritus and erythema, with reduction of eczematous inflammation. Importantly, the underlying porokeratotic lesions demonstrated reduction of area or peripheral scaling, indicating that the observed clinical improvement primarily reflected suppression of inflammatory activity rather than modification of the epidermal differentiation disorder itself.

This interpretation is further supported by RISH findings demonstrating cytokine expression consistent with reactive immune activation. While JAK inhibition has been explored in a range of inflammatory dermatoses, this case highlights the potential utility of RISH in distinguishing secondary inflammatory signaling from primary disease pathology in porokeratosis. Collectively, these findings suggest that JAK1 inhibition may represent a targeted therapeutic option for symptomatic inflammatory manifestations of eruptive pruritic papular porokeratosis, while emphasizing that such therapy should not be considered disease-modifying for porokeratosis itself.

## Conflicts of interest

None disclosed.
